# Innovative Strategies in the Treatment of Unresectable Locally Advanced NSCLC: A Focus on Radiation Therapy

**DOI:** 10.3390/cancers18183061

**Published:** 2026-09-21

**Authors:** Marco Galaverni, Marzia Cerrato, Niccolò Giaj-Levra, Patrizia Ciammella, Fabio Arcidiacono, Paola Anselmo, Federico Colombo, Valeria Dionisi, Giampaolo Montesi, Paolo Borghetti, Alessio Bruni, Marcello Tiseo, Nunziata D’Abbiero, Umberto Ricardi, Nicola Simoni

**Affiliations:** 1 Department of Radiation Oncology and Radiosurgery, Azienda Ospedaliera Universitaria, 43126 Parma, Italy; fcolombo@ao.pr.it (F.C.); ndabbiero@ao.pr.it (N.D.); 2 Radiation Oncology Unit, Department of Oncology, AOU Città della Salute e della Scienza di Torino, 10126 Turin, Italy; mrz.cerrato@gmail.com; 3 Department of Advanced Radiation Oncology, IRCCS Ospedale Sacro Cuore Don Calabria, Cancer Care Center, Negrar di Valpolicella, 37024 Verona, Italy; niccolo.giajlevra@sacrocuore.it; 4 Radiation Oncology Unit, Azienda USL-IRCCS di Reggio Emilia, 42100 Reggio Emilia, Italy; patrizia.ciammella@ausl.re.it; 5 Radiotherapy Oncology Centre, “S. Maria” Hospital, 05100 Terni, Italy; fabio.arcidiacono@uslumbria2.it (F.A.); paola.anselmo@uslumbria2.it (P.A.); 6 Department of Radiation Oncology, University of Verona Hospital Trust, 37126 Verona, Italy; valeria.dionisi@aovr.veneto.it; 7 Radiation Oncology Unit, Radiologic Sciences Department, AST Pesaro Urbino, 61121 Pesaro, Italy; giampaolomontesi@gmail.com; 8 Radiation Oncology Department, ASST Spedali Civili and University of Brescia, 25123 Brescia, Italy; paolobor82@yahoo.it; 9 Radiotherapy Unit, Department of Oncology and Ematology, University Hospital of Modena, 41124 Modena, Italy; bruni.alessio@aou.mo.it; 10 Department of Medicine and Surgery, University of Parma, 43126 Parma, Italy; marcello.tiseo@unipr.it; 11 Medical Oncology Unit, University Hospital of Parma, 43126 Parma, Italy; 12 Department of Oncology, University of Turin, 10126 Turin, Italy; umberto.ricardi@unito.it

**Keywords:** radiotherapy, non-small cell lung cancer, unresectable locally advanced, concurrent chemo-radiotherapy, immunotherapy, stereotactic ablative body radiotherapy, organ-at-risk sparing, risk-adapted de-escalation

## Abstract

Unresectable locally advanced non-small cell lung cancer (LA-NSCLC) remains a therapeutic challenge, despite the paradigm shift established by the PACIFIC trial (concurrent chemoradiation followed by Durvalumab consolidation). A substantial number of patients still experience disease progression and significant toxicity. This narrative review explores innovative radiation therapy (RT) strategies and their integration with systemic therapy. Key innovations analyzed include: 1. Systemic Therapy Intensification: Induction chemo-immunotherapy or novel multi-agent consolidation regimens show promise, but managing increased toxicity remains critical. The integration of DNA damage response inhibitors with RT is also being actively explored to maximize radiosensitization. 2. RT Dose and Volume Modulation: Recent data suggests that dose escalation via highly conformal hypofractionation or stereotactic ablative body radiotherapy (SABR) may enhance efficacy, particularly when combined with immunotherapy. Conversely, risk-adapted de-escalation of dose or volumes is studied to reduce immunosuppression and treatment-related toxicity, allowing more patients, including the elderly and frail, to complete consolidation. 3. Organ-at-Risk (OAR) Sparing: Advanced techniques like intensity-modulated radiation therapy (IMRT), adaptive RT (ART), and proton beam therapy (PBT) are crucial for minimizing dose to OARs and new substructures, such as specific cardiac subregions and lymphoid tissues. This OAR sparing is strongly correlated with improved OS and reduced immunosuppression. In conclusion, this narrative review focuses on the future of LA-NSCLC treatment, which is moving toward personalized, precision RT, embracing both intensification and thoughtful de-escalation, supported by synergistic systemic agents, to maximize curative potential and minimize toxicity.

## Simple Summary

Unresectable locally advanced NSCLC management is evolving beyond the PACIFIC standard. This review examines innovative strategies to improve outcomes and manage toxicity. Key focus areas include: 1. Systemic Integration: This focus area explores induction chemo-immunotherapy, novel consolidation regimens and DNA damage response inhibitors combined with RT. 2. RT Optimization: Recent data supports dose escalation (hypofractionation/SABR) for improved efficacy. Conversely, risk-adapted de-escalation reduces toxicity and radiation-induced lymphopenia. 3. Advanced Organ-at-Risk Sparing: Modern RT techniques prioritize sparing critical cardiac and lymphoid substructures, which correlates with better overall survival. The future involves personalized RT, balancing intensification and strategic de-escalation for curative, less toxic therapy.

## 1. Introduction

Unresectable locally advanced non-small cell lung cancer (LA-NSCLC) represents a significant therapeutic challenge. For decades, the standard of care for these patients has been concurrent chemoradiation therapy (CRT), a multimodal approach that offers the best chance of survival. However, it has been associated with significant toxicities and modest outcomes, with historical 5-year overall survival (OS) rates of around 15–30% [1].

The treatment landscape for LA-NSCLC has been redefined by the PACIFIC trial [2,3,4,5,6,7,8]. In this phase 3, randomized, double-blind, placebo-controlled trial, patients who did not experience progression after platinum-based concurrent CRT were randomized to receive consolidation immunotherapy with Durvalumab, a PD-L1 inhibitor, or a placebo for up to 12 months. The results were practice-changing, demonstrating a notable improvement in the primary endpoint of progression-free survival (PFS) and, subsequently, a significant and durable OS benefit of Durvalumab consolidation therapy (estimated 5-year OS 42.9% for Durvalumab compared to 33.1% for the placebo; HR 0.72, 95% CI 0.59–0.89).

However, the PACIFIC trial must not be seen as the end of the line. Despite these advances, a substantial number of patients treated with concurrent CRT and consolidation immunotherapy still experience disease progression, and the risk of severe toxicities, particularly pneumonitis, remains a critical concern. Furthermore, patients with NSCLC harboring a certain actionable oncogenic alteration (AGA) may benefit more from target therapy than from an immunotherapy-integrated regimen, as demonstrated by the recently published LAURA trial for EGFR-mutant NSCLC [9]. Apart from patients with AGAs, many complex questions regarding the optimization of treatment sequences, the prevention and management of potential adverse events, the optimization of patient selection and the identification of biomarkers predictive of response remain unanswered. This narrative review aims to explore the landscape of innovation in radiation therapy for LA-NSCLC beyond the established PACIFIC standard. It will examine emerging strategies to integrate radiation and immunotherapy in the different settings, as well as critical efforts to optimize the radiation therapy component itself. This includes novel approaches to dose and volume modulation, ranging from dose escalation to thoughtful de-escalation in selected patient groups, as well as advanced techniques to protect critical organs at risk, such as the heart and immune cell populations. Finally, the integration of radiation therapy with other innovative drugs will be reviewed, as we move towards a more personalized and effective therapeutic future for patients with LA-NSCLC.

## 2. Methods

This article presents a structured narrative review with the objective of exploring the landscape of innovation in radiation therapy (RT) for unresectable locally advanced non-small cell lung cancer (LA-NSCLC), looking beyond the established PACIFIC standard. We specifically targeted and integrated the literature evaluating the role of RT in this clinical scenario. Considering the objective to synthesize a broad spectrum of emerging clinical approaches, ranging from novel systemic integrations to complex dosimetric optimizations, a qualitative integration of evidence was selected over a formal meta-analysis. The search scope was meticulously delineated to identify clinical trials and studies addressing the integration of RT with intensified systemic therapy, dose and volume modulation strategies, and advanced techniques for organ-at-risk (OAR) sparing.

To ensure comprehensive coverage of recent clinical advancements, a structured literature search was conducted using the PubMed/MEDLINE, Scopus, and Web of Science databases to identify post-PACIFIC publications from January 2023 to December 2025. Recent publications and trial updates from conferences such as ASTRO, ASCO, ESTRO and ESMO were also included. Search terms combining controlled vocabulary and free-text keywords related to, among others, “radiotherapy”, “non-small cell lung cancer”, “unresectable”, “locally advanced”, “concurrent chemo-radiotherapy”, “sequential chemo-radiotherapy”, “immunotherapy”, “immune checkpoint inhibitors”, “PD-L1 inhibitor”, “hypofractionated radiotherapy”, “stereotactic ablative body radiotherapy”, “stereotactic body radiation therapy”, “organ-at-risk sparing”, “risk-adapted”, “de-escalation”, “radiation-induced lymphopenia”, “adaptive radiotherapy” and “proton beam therapy” were employed. Boolean operators were used to refine the search strategy. Data extraction was performed manually, and only English-language articles were considered. Following an initial assessment by screening titles and abstracts, the studies were reviewed in full and selected for originality and relevance to the review’s broad scope. Screening and selection of all studies were conducted independently by two authors, with any disagreements resolved through discussion. Findings were categorized into predefined thematic domains and organized for narrative synthesis.

## 3. RT and IO Beyond PACIFIC Regimen

The treatment landscape for LA-NSCLC has been profoundly transformed by the introduction of immune checkpoint inhibitors in the context of consolidation strategies following CRT, as established by the PACIFIC trial [2]. Three large real-world cohorts have demonstrated the efficacy and safety of the PACIFIC regimen in clinical practice, confirming the robustness of this therapeutic paradigm [3,4,5]. Nevertheless, questions remain regarding the timing and potential intensification of immunotherapy in this setting, particularly in relation to its integration with CRT.

Two randomized clinical trials have further validated the efficacy of immunotherapy as a consolidation treatment, focusing on maintenance duration. Specifically, PACIFIC-5 compared the use of Durvalumab until progression or toxicity versus a placebo [6], while GEMSTONE-301 compared Sugemalimab for 24 months versus a placebo [7]. However, the positive results of these studies are limited by the standard comparator arm, which is no longer considered current.

The PACIFIC study only offered immunotherapy consolidation to patients treated with concomitant CRT, regardless of PD-L1 expression [8]. Many regulatory agencies have opened up access to immunotherapy even after sequential CRT regimens. The non-randomized phase II PACIFIC-6 trial confirmed that, although outcomes were less satisfactory, the sequential regimen can also be adopted in all cases in which concomitant CRT is not feasible, particularly due to patient frailty, comorbidities, or an excessive treatment volume [10].

Although consolidation with Durvalumab after concurrent CRT remains the gold standard, several randomized and signal-seeking studies have tested the possibility of improving outcomes by modifying the timing of immunotherapy or by intensifying consolidation by combining different agents. The phase III PACIFIC-2 study evaluated Durvalumab administered concurrently with CRT but did not demonstrate a statistically significant advantage in terms of progression-free survival (PFS) compared to CRT alone [11]. This result highlights that immediate and concomitant immune checkpoint blockade is not yet clearly superior to the sequential PACIFIC regimen. Similarly, other studies such as CheckMate 73L, ECOG-ACRIN EA5181 and KEYVIBE-006, which used Nivolumab, Durvalumab and Vibostolimab concurrently with CRT, respectively, failed to demonstrate any benefit [12,13,14]. The only study where the concomitant use of other PD-1/PD-L1 inhibitors with CRT produced encouraging signs of sustained antitumor activity is the single-arm KEYNOTE-799 study with Pembrolizumab [15]. This indicates that selected combinations may be effective and well tolerated, although definitive randomized comparisons are still required.

Promising data from the COAST study (Durvalumab ± Oleclumab or Monalizumab) suggested improved objective responses and PFS when Durvalumab was combined with anti-CD73 or NKG2A antibodies, prompting further phase III efforts (the ongoing PACIFIC-9 study) to formally test multidrug consolidation [16]. It should be noted that not all maintenance phase enhancement strategies with drug combinations have been successful, as recently highlighted by the SKYSCRAPER-003 study that showed the non-superiority of Atezolizumab and Tiragolumab compared to the current standard of care [17].

Recently, several phase II studies have explored the combination of chemo-immunotherapy followed by radiotherapy (with or without chemotherapy and/or immunotherapy) and further immunotherapy maintenance in patients with unresectable stage III NSCLC. The APOLO trial tested an induction regimen with Atezolizumab plus chemotherapy, followed by concurrent CRT and maintenance Atezolizumab [18]. In the intention-to-treat population, the 12-month PFS and OS were 68.4% and 86.8%, respectively, while in the per-protocol population, these figures rose to 78.1% and 90.6%. Grade 3–4 adverse events (AEs) occurred in 23.7%, 34.2% and 13.2% in the induction phase, concurrent CRT phase and maintenance phase, respectively. Only a single treatment-unrelated grade 5 AE was reported. Similarly, the PACIFIC-BRAZIL study investigated a regimen with induction Durvalumab in combination with chemotherapy, followed by concurrent CRT and Durvalumab and subsequent Durvalumab as maintenance [19]. The twelve-month PFS and OS were 68.1% and 81.2%, respectively. However, high rates of grade ≥ 3 treatment-related AEs (82%) were reported, particularly during the induction and concurrent phases. In particular, seven grade 5 events occurred, two of which were considered treatment-related. Finally, Tang et al. evaluated induction Tislelizumab plus chemotherapy followed by radiotherapy (RT) and consolidation Tislelizumab [20]. This study reported an encouraging 12-month PFS of 86.7%, with an objective response rate of 86.7% and a disease control rate approaching 97%. Adverse events were consistent with prior reports, with grade 3–4 events occurring in 20% of patients, though no grade 5 AEs were observed.

Overall, these findings suggest that a combination of induction chemo-immunotherapy, radiation therapy (whether combined with chemoimmunotherapy or not), and immunotherapy maintenance may offer significant survival benefits in LA-NSCLC. However, the toxicity burden remains significant, particularly in regimens involving concurrent CRT and prolonged immunotherapy exposure, highlighting the need to refine patient selection and optimize treatment sequencing.

## 4. Beyond Standard RT

### 4.1. Dose Escalation Through Hypofractionation

Oncological outcomes for patients with LA-NSCLC treated with concurrent CRT followed by immunotherapy remain poor. The primary tumor represents the most common site of non-metastatic failure, with a cumulative loco-regional failure (LRF) rate of 20–40%, including synchronous loco-regional and systemic failure [1,2,21]. As LRF remains a critical challenge even in the PACIFIC era, there is an ongoing interest in exploring novel strategies to enhance survival by improving local control.

Dose escalation for NSCLC has produced mixed results. Some studies have indicated potential benefits in terms of local control and survival, while others have highlighted increased toxicity with no significant improvement in survival. During the pre-immunotherapy era, the RTOG 0617 trial found that dose escalation with conventionally fractionated RT up to 74 Gy was associated with a lower median OS of 20.3 months compared to 28.7 months for 60 Gy, with higher rates of severe toxicities [1]. One reasonable postulation to explain the detrimental effect on survival of dose escalation in the RTOG 0617 trial is that the presence of excess cardiopulmonary toxicity from large mediastinal fields led to an elevated risk of death, while the increase in the biologically effective dose (BED) by conventional fractionation was insufficient to improve oncological outcomes. Thus, 60 Gy in conventionally fractionated RT (CFRT) has remained the established standard of care since then.

The use of hypofractionated RT (HFRT) as a regimen for dose escalation has already been explored in the pre-immunotherapy era (Table 1). A phase III randomized clinical trial failed to demonstrate an improvement in the PFS and OS with HFRT (60 Gy in 15 fractions) compared to the standard of care (60 Gy in 30 fractions) for patients with stage II-III NSCLC ineligible for concurrent CRT [22]. However, there was an almost three-fold increase in grade 2 toxicity in the hypofractionated arm. Although there were no differences in grades 3–5 side effects, the increase in grade 2 adverse events may be of concern for such a frail patient population.

**Figure 1:**
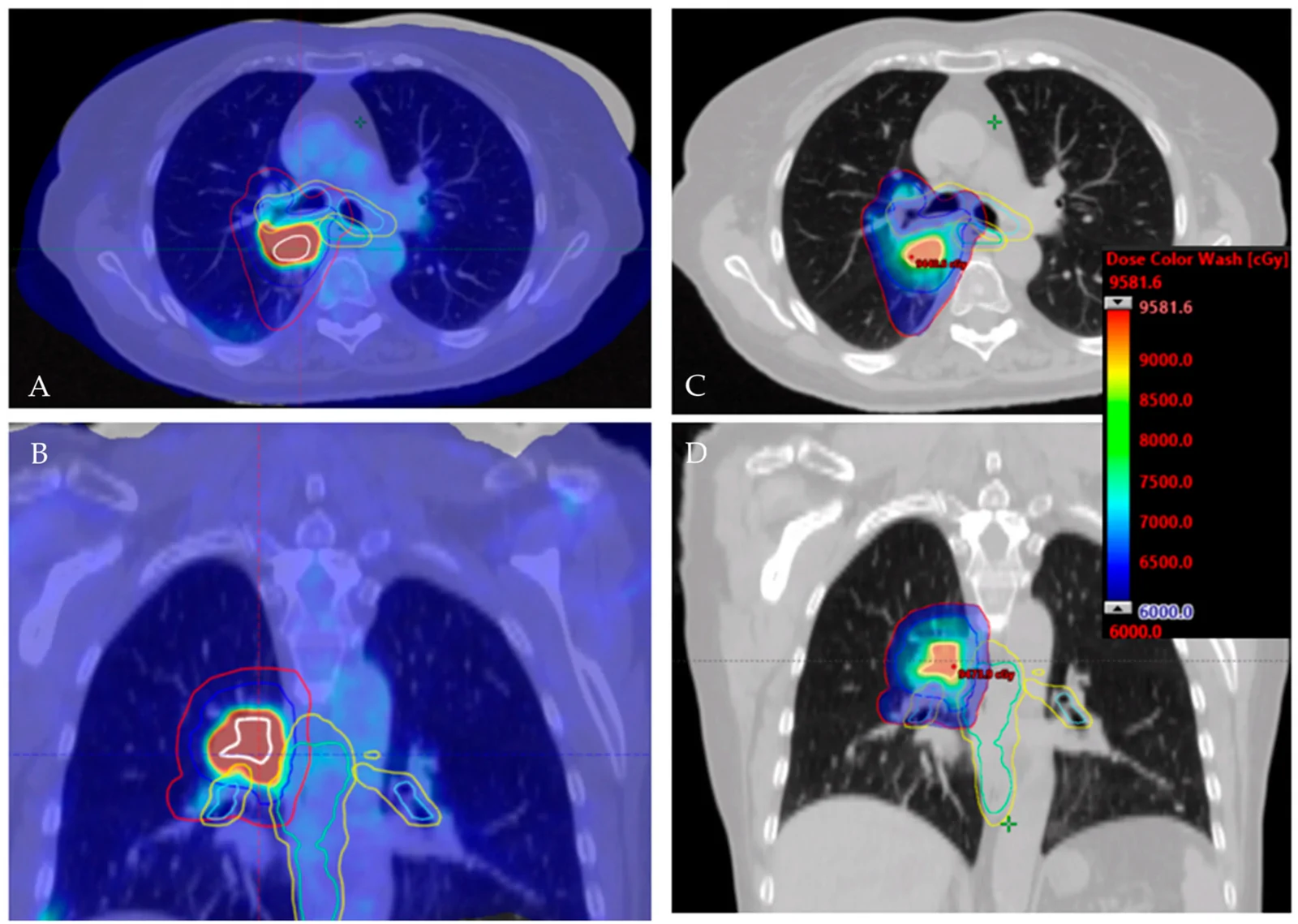
(**A**,**B**) Right lower lobe, unresectable LA-NSCLC, demonstrated on non-contrast four-dimensional radiotherapy planning CT. Co-registered PET-FDG in planning position is superimposed. Red line: planning tumor volume (PTV); blue line: clinical target volume (CTV); white line: PET-FDG-based dose-escalated subvolume (50% of SUVmax was chosen as the threshold for volume generation); cyan line: main bronchi; green line: esophagus; yellow line; planning organ-at-risk volumes of esophagus and bronchi. (**C**,**D**) Treatment plan with dose superimposed. Volumetric arc therapy (VMAT) was the technique of choice. Through simultaneous integrated boost (SIB), 60 Gy, 66 Gy and 90 Gy were prescribed to PTV, CTV and PET-FDG subvolumes, respectively.

In patients fit for concurrent CRT, hypofractionation has often been explored in a PET-guided approach, allowing for a more targeted dose delivery and thereby minimizing harm to surrounding tissues (Figure 1). Encouraging results came from the Chinese phase III trial (CRTOG1601), which randomized patients to receive an adaptive PET-based dose-escalated arm to a dose ≥ 66 Gy versus 60 Gy in CFRT [23]. The primary objective was met with superior OS and PFS in the experimental arm, with no increase in toxicity. However, the increased delivered doses did not always translate into better outcomes, as seen in the PET-Boost [24] and PET-Plan [25] trials, which reported high loco-regional control (LRC) rates at the cost of high severe toxicity for both whole and tumor subvolume experimental arms. Disappointing results were also seen in the RTOG 1106/ACRIN 6697 trial [26], a randomized phase II study evaluating the superiority of a PET-based HFRT up to an 80.4 Gy arm versus 60 Gy in CFRT. Although no differences in G2-G3 adverse events were seen, the primary objective was not met, with similar loco-regional control (LRC) in the standard and experimental arms.

More recently, promising results have come from two HFRT PET-based trials which included immunotherapy consolidation in some patients (Table 2). RTEP7-IFCT-1402 was a randomized phase II trial that met its primary endpoint: adaptive HFRT up to 74 Gy in 33 fractions was associated with higher LRC than the standard arm of 66 Gy in CFRT, without any difference in toxicity between the two arms [27]. NARLAL-2, an international multicenter phase III trial, randomized patients between heterogeneous dose escalation through HFRT (aiming at mean doses of 95 Gy and 74 Gy in 33 fractions for primary tumors and node tumors, respectively) and 66 Gy in CFRT. Early results showed superior LRC in the experimental arm (primary endpoint), without an increase in early toxicity, and a promising OS rate [28]. In addition, the ALLSTAR study (a prospective registry aimed at validating the PACIFIC regimen in daily practice in Austria) has confirmed the benefit of Durvalumab consolidation for OS and PFS, as well as significantly better LRC with RT dose escalation > 66 Gy (2-year LRC rates: 82% versus 62% with standard doses, respectively) [29].

**Table 1:** Summary of relevant dose escalation trials for LA-NSCLC in pre-immunotherapy era.

Trial, Year	PET-Plan, 2020 PET-Based Target, Dose-Escalated Arm [25]	PET-Plan, 2020 Conventional Target, Dose-Escalated Arm [25]	CRTOG 1601, 2020 Dose-Escalated Arm [23]	Iyengar et al., 2021 Hypofractionated Dose-Escalated RT Arm [22]	PET-Boost, 2023 Whole-Tumor Dose-Escalated Arm [24]	PET-Boost, 2023 Subvolume Dose-Escalated Arm [24]	RTOG 1106/ACRIN 6697, 2021 PET-Guided Dose-Escalated Arm [26]
Design	Phase II	Phase II	Phase III	Phase III	Phase II	Phase II	Phase II
RT technique	53% IMRT 46% 3DCRT 1% missing	55% IMRT 44% 3DCRT 1% missing	NS	NS	61% IMRT 29% VMAT	60% IMRT 40% VMAT	NS
Number of patients	106	99	113	50	54	53	84
Median age (years)	65, 5	64	NS	71	65.5	69	65
Median follow-up (months)	29	29	NS	8.7	61	61	43.2
Stage IIIA/IIIB/other (%)	39/54/8	37/55/8	NS	72/28 °	56/35/9	62/23/15	54.8/45.2/0
Median BED10 (gray)	80.8 *	78.4 *	NS	84	101.6 *	112.2 *	87.8 *
LC	1-y 86% 2-y 82% 4-y 78%	1-y 75% 2-y 65% 5-y 62%	NS	2-y 86%	1-y 97% 1.5-y 89%	1-y 91% 1.5-y 82%	2-y 54.6% ^
Median PFS (months)	11	10	15.1	6.4	10	11.5	13.8
Median OS (months)	29	37	44.6	8.2	18	17	31.2
≥G3/G5 lung toxicity (%)	26/6.3	22/3.4	NS/NS	28/4	19/15	32/21	6.9/5.9

LA-NSCLC, locally advanced non-small cell lung cancer; NS, not specified; VMAT, volumetric arc therapy; IMRT, intensity-modulated radiation therapy; BED10, biologically effective dose at α/β 10 Gy; LC, local control; PFS, progression-free survival; OS, overall survival; G, grade. ^ Intrathoracic control; * mean dose; ° stage III/non-stage III.

**Table 2:** Summary of relevant dose escalation trials for LA-NSCLC in immunotherapy era (including Durvalumab arm of PACIFIC trial for comparison).

Trial, Year	PACIFIC, 2017 60 Gy + Durvalumab Arm [8]	NARLAL-2, 2025 Dose-Escalated Arm [28]	RTEP7-IFCT-1402, 2024 Dose-Escalated Arm [27]
Design immunotherapy consolidation (%)	Phase III 100	Phase III 20	Phase II 48
RT technique	NS	100% IMRT or VMAT	81% IMRT 19% 3DCRT
Number of patients	476	173	68
Median age (years)	64	66	62
Median follow-up (months)	34.2	50.8	45
Stage IIIA/IIIB/other (%)	52.9/44.5/2.5	61.3/34.1/4.6	59/40/1
Median BED10 (gray)	NS	102.9	90.6
LC	61.5% at a median follow-up of 25.2 m ^	1 y 69% ° 2 y 61% ° 4 y 47% °	15 m 77.6%
Median PFS (months)	16.9	NA	22.3
Median OS (months)	47.5	51.6	NR
≥G3/G5 lung toxicity (%)	12/4	7.3/1.7	4.4/1.5

LA-NSCLC, locally advanced non-small cell lung cancer; NS, not specified; VMAT, volumetric arc therapy; IMRT, intensity-modulated radiation therapy; BED10, biologically equivalent dose at α/β 10 Gy; LC, local control; PFS, progression-free survival; OS, overall survival; G, grade; NA, not available; NR, not reached. ^ intrathoracic control; ° loco-regional control.

Undoubtedly, HFRT represents a rational strategy to deliver higher and even ablative doses in LA-NSCLC, providing promising loco-regional control and survival rates. However, to date, data in support of HFRT are limited by the potential for toxicity, and the optimal radiotherapy schedule and prescribing dose remain unanswered questions. In addition, despite recent promising data on the synergy between dose-escalated HFRT and consolidation immunotherapy, the optimal timing of combination therapies, integration with other systemic agents, and patient selection criteria remain areas of ongoing research in LA-NSCLC.

### 4.2. Dose Escalation Through Stereotactic Ablative Body Radiotherapy (SABR)

Ultra-hypofractionated regimens, also known as stereotactic body radiation therapy (SBRT) or stereotactic ablative body radiotherapy (SABR), have recently emerged as a possible approach in LA-NSCLC, increasing biologically effective doses (BEDs) without lengthening treatment time [30]. Recent technological advances in RT have made it possible to administer a few fractions (typically from three to eight fractions) of high external beam doses to the tumor, sparing the surrounding healthy tissues by a rapid fall in the dose outside the target. SABR represents the standard of care for inoperable early-stage NSCLC, but there are limited data in LA-NSCLC, with major concerns about safety in central and ultra-central lesions [31,32,33,34,35].

**Figure 2:**
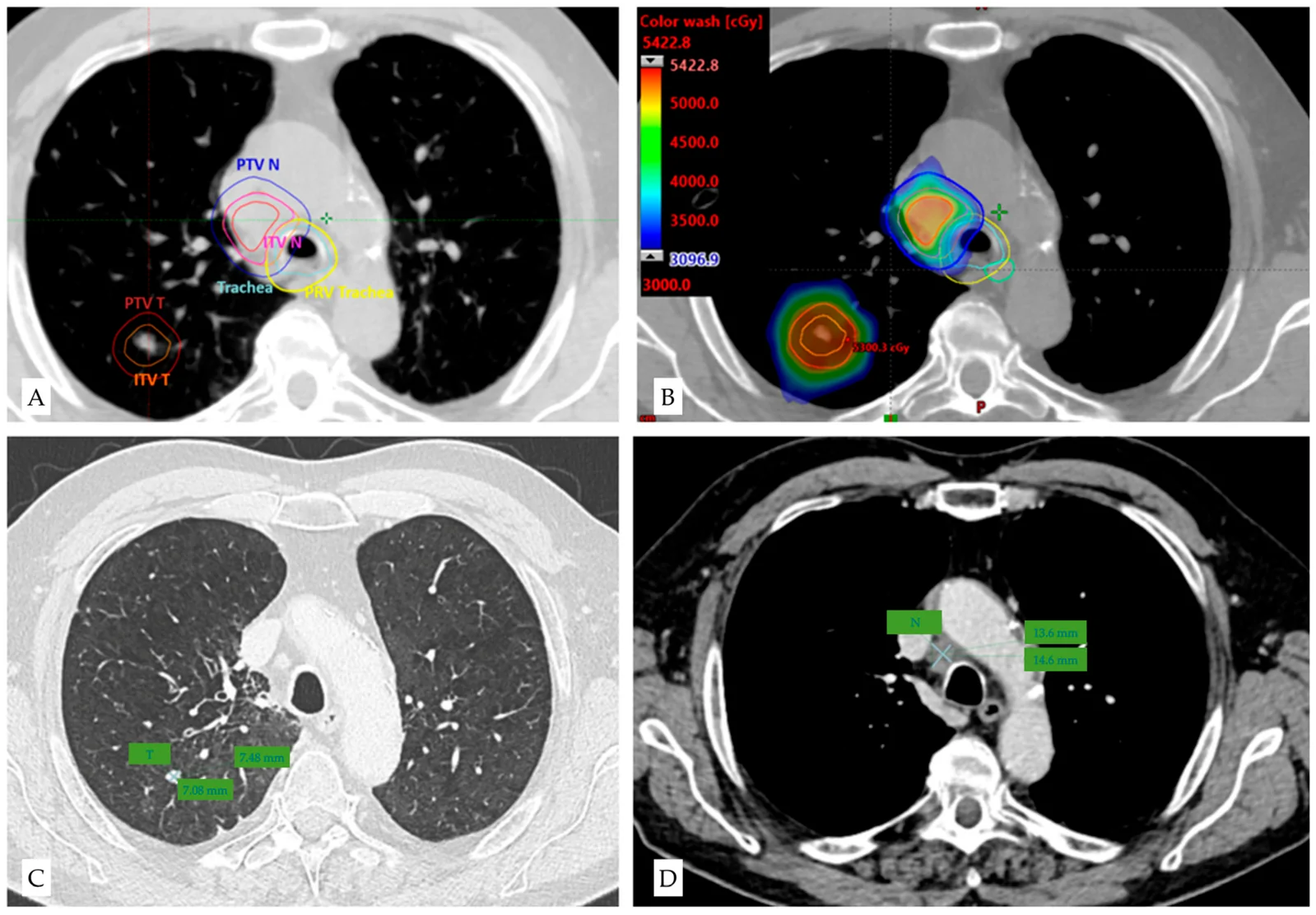
(**A**) Right upper lobe, unresectable LA-NSCLC, demonstrated on non-contrast four-dimensional radiotherapy planning CT. PTV N, planning tumor volume of involved node; ITV N, internal target volume (ITV) of involved node; PTV T, PTV of primary tumor; ITV T, ITV of primary tumor. (**B**) Treatment plan with dose superimposed. Stereotactic ablative body radiotherapy (SABR) was the technique of choice. Through the simultaneous integrated boost (SIB) technique, 30 Gy, 40 Gy, and 50 Gy were prescribed to PTV N, ITV N, PTV T and a subvolume of ITV N, respectively. (**C**,**D**) A diagnostic CT 9 months after SBRT demonstrated the marked partial response of the primary tumor and involved node.

To date, three main approaches have been investigated for the use of SABR in LA-NSCLC patients:

1. As an exclusive approach to both the primary tumor (T) and involved mediastinalnodes (N) (Figure 2);
2. As a boost to residual disease following conventional RT or hypofractionated RT;
3. As a boost to the peripheral T in combination with conventional fractionated RT to the mediastinal N.

Few studies have investigated the effectiveness and safety of SABR to the T and N for unresectable LA-NSCLC patients (Table 3) [36,37,38,39,40,41,42,43]. The START-NEW-ERA phase II trial reported the largest series with the longest follow-up [36]. Fifty LA-NSCLC patients received median doses of 45 Gy and 40 Gy in five daily fractions based on FDG-PET to primary tumors and regional nodes, respectively, with the implementation of simultaneous integrated boost (SIB) and simultaneous integrated protection (SIP). After a median follow-up of 72 months, median local recurrence-free survival (LRFS) was not reached, the 5-year PFS was 26%, and the median OS was 53 months. No patients experienced G ≥ 3 acute or late adverse events. In a post hoc analysis, adenocarcinoma histology and a SABR dose ≥ 40 Gy were significant predictors of better LC, while the use of SIP did not influence LC [37].

The favorable efficacy observed in the START-NEW-ERA trial, despite the absence of chemotherapy (CHT) in 46% of patients, suggests that SABR could represent a promising treatment strategy for patients who are not suitable for standard therapy due to age or other health conditions. This hypothesis is corroborated by a recent meta-analysis conducted by Viani et al. assessing the role of SABR in 268 patients with unresectable LA-NSCLC treated with definitive SABR to both the T and N in five daily fractions. With a reported 1-year LC of 80%, the incidence of G ≥ 3 toxicity was 5%, with 1.7% treatment-related mortality [44]. In addition, a linear correlation between OS and LC was described.

SABR has also been explored as a boost following conventional or hypofractionated RT. Wu et al. recently reported a dose escalation study including 28 LA-NSCLC patients treated with adaptive SABR of 25 Gy (low-dose group), 30 Gy (intermediate-dose group), or 35 Gy (high-dose group) in five fractions to residual metabolically active disease on FDG-PET after 40 Gy in 10 fractions of CRT, without immunotherapy consolidation [45]. After a median follow-up of 18 months, the 2-year LC and OS were 74.1% and 30%, 85.7% and 76.2%, and 100% and 55.6% for the low-, intermediate- and high-dose cohorts, respectively. The adaptive SABR boost showed promising LC, suggesting that increasing the BED may enhance intra-thoracic control. However, two deaths (22% of patients) occurred in the high-dose cohort, with the intermediate-dose regimen appearing to offer the best balance between efficacy and safety.

More recently, Heinzerling et al. reported the outcomes of 61 patients with LA-NSCLC treated with SABR (50–54 Gy in three–five fractions) to peripheral T (central tumors within 2 cm of the involved N were excluded), followed by conventionally fractionated CRT to the N, with consolidative immunotherapy administered in the majority of cases [46]. After a median follow-up of 29.5 months, the 2-year LC rate of the T was 96.3%, highlighting the potential benefit of SABR-based dose escalation. The 1- and 2-year PFS rates were 62.8% and 50.1%, respectively, while the 1-year and 2-year OS rates were 80.3% and 68.0%, respectively. Despite the predominance of peripheral T (90%), G ≥ 3 adverse events occurred in 18% of patients, with treatment-related discontinuation and death observed in 8% and 7% of cases, respectively. This treatment strategy (SABR to the T combined with conventionally fractionated RT to the N) is currently under investigation in the phase III LU-008 trial [47].

Collectively, the above reported findings support a role for SABR in LA-NSCLC. Notably, delivering a biologically effective dose (BED) exceeding 100 Gy could improve oncological outcomes by maximizing local tumor control. In addition, the sharp dose fall-off can spare adjacent healthy tissues, potentially minimizing adverse events and treatment-related immunosuppression. Furthermore, the reduced overall treatment time could enable seamless integration with systemic therapies. However, several clinical challenges warrant careful consideration. The risk of severe toxicity to ultra-central structures, such as the trachea, main bronchi, and esophagus, demands stringent constraints. Furthermore, the optimal dose prescription for regional lymph nodes is controversial, and complex dose summation issues may arise when combining SABR to the primary tumor with conventional radiotherapy (CFRT) to the mediastinum. In this context, emerging technologies, such as online adaptive radiotherapy and real-time tracking of the tumor, could further optimize SABR delivery and patient outcomes.

### 4.3. Tumor Dose and Volume De-Escalation

While the PACIFIC regimen has transformed prognosis, it has also introduced new challenges. Pulmonary toxicity, such as pneumonitis, represents a significant concern with both RT and immunotherapy [8,48,49,50]. Previous CRT-related toxicity is a significant barrier to Durvalumab eligibility for some patients. Therefore, lower RT doses and/or more conformal treatments could reduce these percentages. Furthermore, the incidence of grade 2 pneumonitis is higher among patients receiving consolidation immunotherapy. In this context, a lower volume of lung receiving 20 Gy (V20) has been shown to reduce this incidence [51]. In the PACIFIC trial, 15.4% of patients discontinued Durvalumab due to adverse events (compared to 9.8% in the placebo group) [8], whereas in the real-world PACIFIC-R study, adverse events led to discontinuation in 16.5% of patients [52].

The rationale for RT dose de-escalation includes reducing the direct side effects of radiation (e.g., esophagitis and pneumonitis), as well as creating a less immunosuppressive tumor microenvironment, while maintaining or even enhancing therapeutic efficacy. A less toxic radiation schedule enables more patients to complete immunotherapy and initiate it earlier after CRT or concomitantly, thereby maximizing their chances of long-term success. De-escalation is currently being explored in multiple and complementary areas:

1. Shrinking the volume of irradiated tissue;
2. Reducing the total radiation dose for low-risk tumors;
3. Reducing the dose for selected patient populations;
4. Reducing the dose to organs at risk or particular organ subregions.

#### 4.3.1. Volume De-Escalation

Several trials investigating the omission of the clinical target volume (CTV) have shown promising results [53,54,55] (Table 4). Notably, Cui et al. conducted a randomized phase II trial in which patients with unresectable stage III NSCLC undergoing intensity-modulated RT (IMRT) were randomly assigned to a study group (CTV omitted under PET-CT guidance) or control group (CTV delineated) [55]. The incidence of radiation-related respiratory events or grade ≥ 3 esophagitis was significantly lower in the study group (11% vs. 29% in the control group), due to the reduced irradiated volumes. The median PFS and OS were similar between the two groups (9 vs. 10.0 months and 31.0 vs. 26.0 months, respectively). Failure patterns were also similar.

In conclusion, omitting the CTV has shown preliminary feasibility, reducing severe radiation-associated adverse events for LA-NSCLC without compromising efficacy [56]. However, more prospective evidence is needed to evaluate this approach in the era of four-dimensional computed tomography (4DCT), image-guided radiation therapy (IGRT), and surface-guided radiation therapy (SGRT).

#### 4.3.2. Tumor Dose De-Escalation

The randomized REPAINT trial recently tested risk-adapted radiation therapy de-intensification for patients with LA-NSCLC receiving CRT [57]. The metabolic tumor volume for the primary tumor and involved nodes was calculated using FDG-PET. Participants were randomly assigned to receive either standard RT (60 Gy in 30 fractions) or dose-painted RT (55 Gy delivered to tumors and lymph nodes with metabolic volumes exceeding 20 cm^3^ and 44–48 Gy to other lesions, all in 20 fractions). The progression-free survival and OS rates were similar across the study arms, while grade 3–4 lymphopenia was reduced in the de-escalated arm.

The SPRINT trial explored the role of risk-adapted radiotherapy in combination with induction and consolidation immunotherapy in LA-NSCLC patients with a PD-L1 tumor proportion score of ≥50% [58]. The prescribed dose was personalized according to the response to induction immunotherapy in a PET-FDG-based, chemotherapy-free approach. Through simultaneous integrated boost (SIB), 55 Gy in 20 fractions was delivered to lesions with a metabolic volume exceeding 20 cm^3^, while 48 Gy in 20 fractions was delivered to smaller lesions. Promising efficacy was observed, with 1-year PFS, 1-year OS and 2-year OS rates of 76%, 92% and 76%, respectively. No cases of severe pneumonitis were reported.

In conclusion, although further studies are needed to confirm these results, risk-adapted radiotherapy dose de-escalation does not appear to compromise clinical outcomes or disease control and has the potential to reduce treatment-related adverse events and immunosuppression.

#### 4.3.3. Selected Patient Population De-Escalation

Elderly and frail patients are underrepresented in clinical trials and may experience higher rates of side effects. In the PACIFIC post hoc analysis, 41.6% of patients aged ≥70 years experienced grade 3–4 adverse events [59]. In addition, a significant proportion of patients with unresectable LA-NSCLC are ineligible for platinum-based concurrent CRT, due to comorbidities, poor performance status, or advanced age. Historically, these patients have been treated with sequential CRT or RT alone, resulting in unsatisfactory outcomes. There is currently no established standard of care for this population. However, acceptable outcomes have been observed in patients treated with Durvalumab following sequential CRT, as evidenced by the PACIFIC-R overall survival analysis (3-year OS of 57.9%) [3]. In addition, preliminary findings from PACIFIC-6 [10] suggest that Durvalumab administered after sequential RT has a comparable safety profile to that observed in PACIFIC following concomitant treatment, and it may therefore represent an alternative strategy for patients who are not candidates for concurrent therapy.

In the near future, chemo-free protocols may be considered, and ongoing trials are evaluating the combination of RT and immunotherapy for patients who are unfit for concurrent CHT [60,61,62,63]. In this context, recent data from the DUART study, which enrolled a frail, real-world reflective population, including patients treated with either definitive RT (60 Gy ±10% or BED-equivalent) or palliative RT (40–54 Gy or BED-equivalent), indicated that RT followed by Durvalumab yields an adverse-event profile broadly comparable to that observed in patients receiving Durvalumab after concomitant CRT [63,64,65]. In this trial, 25.5% of patients discontinued Durvalumab due to adverse events (most commonly pneumonitis), whereas 21.6% discontinued due to disease progression. By comparison, the discontinuation rate for adverse events in the PACIFIC trial was 15.4%. Efficacy signals were encouraging relative to historical RT-only series: the median OS was 21.1 months (12-month OS, 64.7%), exceeding the 8–11 months typically reported for RT alone in chemotherapy-ineligible patients. Similarly, the Japanese single-arm, prospective, open-label, multicenter phase II trial SPIRAL-RT study enrolled patients who are ineligible for concurrent chemoradiotherapy to receive RT (54–66 Gy) followed by Durvalumab for up to 12 months [66]. Reported outcomes in 33 patients included 1-year PFS and OS rates of 39.1% and 71.7%, respectively. The most common adverse event was radiation pneumonitis (51.5%), and 23 patients (71.8%) discontinued treatment, mostly for disease progression.

Reports of excessive pulmonary toxicity with concomitant thoracic RT and Durvalumab largely derive from the single-arm study by Zhang et al., which was early-terminated due to a high incidence of grade ≥ 3 treatment-related adverse events (a cumulative rate of 60%, with two fatal pulmonary events) [67]. However, other studies involving elderly and frail patients have suggested both feasibility and acceptable tolerability. The TRADE-hypo trial was an open-label, phase II study that evaluated the safety and efficacy of concurrent Durvalumab in elderly and frail patients receiving either hypofractionated RT (55 Gy in 20 fractions of 2.75 Gy, using a stop-and-go regimen) or conventional fractionation (60 Gy in 30 fractions) [68]. An increased incidence of pneumonitis (including 16.7% grade 5 events) was observed in the hypofractionated cohort during the interval period, leading to the closure of recruitment in that arm. Taken together, these findings suggest that the concurrent administration of Durvalumab with RT may pose significant safety risks in patients ineligible for chemotherapy, necessitating careful patient selection and regimen choice.

Currently, the DEDALUS trial (NCT05128630) is investigating a sequential approach that combines induction chemo-immunotherapy, accelerated hypofractionated RT (HFRT; 45 Gy/15 fractions) plus Durvalumab, and Durvalumab consolidation for up to 12 cycles in patients unsuitable for concurrent chemoradiotherapy. Early reports indicated acceptable tolerability (28% of patients experienced at least one grade 3–4 possibly related adverse event), with most adverse events attributable to the chemotherapy component [69].

In clinical practice, exclusive hypofractionated radiotherapy may be used in elderly patients with multiple comorbidities to achieve cytoreduction or palliation when other approaches are not feasible [70]. Indeed, it offers a meaningful advantage by shortening the overall treatment time, which is particularly beneficial for patients with limited mobility. However, the impact of patient frailty and/or age on both tumor control and treatment-related toxicities remains to be defined. De-escalation strategies that simultaneously enhance outcomes warrant a reassessment of traditional standards. This is a multidisciplinary effort that must be tested in clinical trials.

#### 4.3.4. Organ-at-Risk Dose De-Escalation

As previously noted, local control remains a major determinant of OS in patients with unresectable LA-NSCLC [71]. Achieving optimal local control requires precise target delineation, supported by high-quality radiological imaging and bronchoscopic staging, to minimize the risk of geographic miss. It is equally essential to identify and spare organs at risk (OARs), which is facilitated by standardized contouring guidelines and modern technological advancements. The European Society for Radiotherapy and Oncology (ESTRO) has provided detailed recommendations to standardize target delineation and optimize radiotherapy workflows [72]. In the context of concurrent CRT, preserving healthy tissues is crucial for both treatment efficacy and tolerability.

Pulmonary sparing is increasingly recognized as essential to reduce adverse events during subsequent maintenance therapy with immunotherapy [2] or targeted systemic agents [9]. The phase III randomized RTOG 0617 trial remains a landmark study which demonstrated that dose escalation to 74 Gy failed to improve outcomes in the pre-immunotherapy era [1]. Subsequent dosimetric analyses have identified the cardiac dose as a major determinant of poor outcomes, with the mean heart dose (MHD) and multiple volumetric parameters consistently emerging as independent predictors of OS [73,74,75,76,77,78].

More recent studies have emphasized the prognostic significance of individual cardiac substructures [79], supporting the implementation of heart-sparing strategies [80]. Atkins et al. identified a left anterior descending coronary artery (LAD) V15 Gy ≥ 10% as an independent predictor of major adverse cardiac events (MACEs) and all-cause mortality (ACM), particularly in patients without coronary heart disease (CHD) [81], a finding which was subsequently validated [82]. In addition, a left ventricle V15 Gy ≥ 1% was associated with an increased risk of MACEs among patients with CHD [82]. In 2022, a cohort study identified a potential association between higher doses to the cardiac conduction system and cardiotoxicity. A maximum dose (Dmax) to the sinoatrial node (SAN) ≥ 20 Gy was associated with an increased risk of atrial fibrillation (AF) in NSCLC patients (aHR = 15.67; *p* = 0.008), and a higher SAN Dmax was associated with poorer OS (aHR = 1.97; *p* < 0.001) in multivariable analysis [83].

The arrhythmogenic substrate responsible for AF is typically located at the junction of the pulmonary veins and the left atrium. A maximum equivalent dose in a 2 Gy fraction (EQD2) of 23 Gy to the heart base (the region encompassing the right atrium, the origin of the right coronary artery, and the ascending aorta) has been proposed as a dose limit for future studies, as higher doses are strongly associated with early mortality [84]. The Northern Ireland Cardiac Health Events After Radiotherapy (NI-HEART) study confirmed these findings, establishing a link between the maximum dose to the heart base (Dmax) and rates of death and cardiac events after adjustment for patient, tumor, and cardiovascular factors. This suggests that the heart base may be considered a potential sub-organ at risk in order to mitigate radiation-related cardiotoxicity. Furthermore, this retrospective analysis found an association with both the left pulmonary veins’ V55 (HR = 1.02; *p* = 0.005) and the right pulmonary veins’ V10 (HR = 1.01; *p* = 0.033) [85].

Based on the association between an excess dose to the top of the heart and reduced OS [85,86,87], as well as on considerations of cardiac physiology [88], the lung cancer team at The Christie NHS Foundation Trust made the clinical decision to introduce a new dose limit for an anatomical region known as the Cardiac Avoidance Area (CAA) [89]. The aim is to improve survival in patients with stage I–III lung cancer treated with curative-intent radiotherapy. The prospective RAPID-RT study is assessing the feasibility and clinical acceptability of a rapid-learning framework that leverages real-world data in order to formally evaluate outcomes and the impact of implementing the new CAA dose constraint [90]. This work has the potential to establish rapid learning as a rigorous, evidence-based approach for continuously optimizing radiotherapy workflows, with the aim of reducing the risk of radiation-induced heart disease (RIHD) and improving OS. However, a number of methodological gaps must be addressed to strengthen the evidence on cardiotoxicity in thoracic radiotherapy. Dose–volume parameters require external validation, and endpoints for cardiac events, which are currently defined heterogeneously, need rigorous validation and standardization. Studies should be adequately powered, with sufficiently long follow-up periods to capture late cardiac outcomes. Analyses must also account for competing risks, including pre-existing cardiac disease and death from non-cardiac causes. Modeling strategies should also mitigate multicollinearity arising from the inclusion of multiple, highly correlated dosimetric variables. Finally, the concurrent or sequential use of systemic therapy in combination with radiotherapy may confound the observed associations, so this must be considered for appropriate study design and statistical analysis adjustment.

Modern radiotherapy techniques, such as intensity-modulated radiotherapy (IMRT) and volumetric modulated arc therapy (VMAT), enable substantial reduction in cardiac exposure while maintaining adequate target coverage. Nevertheless, uniform delineation protocols and the systematic integration of cardiac constraints are necessary to ensure reproducibility and optimize patient safety. Similarly, sparing of the pulmonary parenchyma is gaining attention, as emerging evidence suggests a strong correlation between preserving lung tissue and a lower incidence of pulmonary adverse events. This has prompted a reassessment of conventional lung dose constraints in the immunotherapy era, with more restrictive thresholds being increasingly considered to minimize pulmonary toxicity [91].

Beyond the lungs and heart, radiation-induced lymphopenia (RIL) has emerged as a critical prognostic factor in NSCLC, with severe lymphopenia consistently associated with poorer clinical outcomes, including OS and PFS [91,92,93,94]. RIL primarily results from the irradiation of hematopoietic and lymphoid structures, including the lungs, heart, spleen, and thoracic vertebrae, which harbor stem cells and lymphocyte progenitors [95]. Image-based analyses have demonstrated that the mean lung dose (MLD), mean heart dose, and thoracic vertebrae V20 strongly correlate with lymphocyte nadir and recovery and act as independent predictors of survival [93]. Cheung et al. identified critical thresholds for these structures, including an MLD > 16.5 Gy and a thoracic vertebrae V20 > 25.6%, both associated with an increased risk of severe lymphopenia [96]. These findings highlight the importance of integrating lymphocyte-sparing approaches into treatment planning and investigating immune-sparing proactive strategies in future clinical trials.

Beyond IMRT, two additional technological strategies warrant consideration: adaptive radiotherapy (ART), guided by computed tomography (CT) or magnetic resonance (MR), and proton therapy. Tumor shrinkage during concurrent CRT is recognized as a favorable prognostic factor for OS [97]. ART enables the replanning of radiotherapy to account for dynamic changes in the target volume and OARs. ART can be implemented in offline, online, or real-time modalities [98], and early clinical experiences have demonstrated its feasibility and potential to reduce toxicity and improve dosimetric precision [99,100,101]. These approaches are being evaluated in prospective studies, providing a framework for potential dose de-escalation strategies while maintaining efficacy.

Proton beam therapy (PBT) offers an additional means of sparing normal tissue due to its physical properties, which enable high doses to be delivered to the tumor with steep dose fall-off beyond the target. Despite its potential, PBT faces challenges in NSCLC due to tissue heterogeneity and respiratory motion. This necessitates precise motion management and treatment margins [102]. Phase II studies have demonstrated the feasibility of PBT combined with concurrent chemotherapy, with low rates of severe radiation pneumonitis [103,104]. PBT also reduces heart exposure to radiation, yielding promising results in terms of local control and tolerability [105]. Furthermore, pencil-beam PBT can reduce the mean heart dose, MLD, and radiation dose to immune cells, which could potentially improve survival outcomes, reduce lymphopenia, and decrease the risk of radiation pneumonitis.

In conclusion, local control remains a main driver of survival in modern radiotherapy for LA-NSCLC. However, if maximizing local control is of paramount importance, it is equally important to implement strategies for careful OAR sparing to ensure that the treatment remains tolerable. In this context, the integration of immunotherapy and targeted therapies has further emphasized the importance of organ preservation in reducing the risk of treatment-related adverse events while maintaining or even enhancing oncological outcomes. Advanced technological approaches, including IMRT, VMAT, ART, and proton therapy, play a central role in minimizing the dose to the heart, lungs, and lymphoid tissues, thereby reducing toxicity and preserving immune function. Early evidence suggests that these strategies may improve survival and treatment tolerability, particularly when combined with immunotherapy. The integration of lymphocyte-sparing planning, adaptive strategies, and advanced particle therapies represents a step toward personalized, precision radiotherapy, optimizing tumor control while minimizing collateral damage.

## 5. RT and Innovative Drug Integration

### 5.1. Biological Rationale and Its Limitations

Beyond the PACIFIC regimen, the most extensively explored strategy is the addition of DNA damage response (DDR) inhibitors, intended to increase the intrinsic radiosensitivity of tumor cells and to amplify the immunogenic consequences of irradiation [106]. In particular, to overcome radiation resistance mechanisms, newer-generation PARP inhibitors are emerging as highly effective, targeted radiosensitizers with strong clinical potential and minimal toxicity [107]. RT kills tumor cells mainly through DNA double-strand breaks (DSBs): PARP inhibitors trap PARP1 on single-strand break intermediates, converting them into replication-associated DSBs; ATM and DNA-PK inhibitors impair DSB signaling and repair; ATR and CHK1 inhibitors abrogate the intra-S and G2/M checkpoints. In parallel, cytosolic DNA generated by irradiation activates the cGAS–STING axis and type I interferon-dependent T-cell priming, a signal that DDR inhibition amplifies, providing the rationale for triple combinations with PD-(L)1 blockade [108,109]. Despite this rationale, a main limitation exists: the therapeutic index depends on a differential dependence of tumor and normal tissue on the targeted pathway, and such differential dependence is far less evident in NSCLC than in the BRCA-mutated cancers in which PARP inhibitors were developed. Homologous recombination deficiency is uncommon in NSCLC, and ATM alterations, present in approximately 5–10% of cases, are frequently missense variants of uncertain functional significance. In the absence of a validated selection biomarker, radiosensitization is therefore expected to be largely non-selective, applying equally to the esophageal mucosa and the lung parenchyma within the high-dose region.

### 5.2. Agents Under Clinical Investigation: Evidence, Safety and Current Status

Among PARP inhibitors, Olaparib provides the most instructive cautionary data. Combined with 66 Gy in 24 fractions, its MTD without cisplatin was only 25 mg once daily, a small fraction of the registered systemic dose, and severe pulmonary adverse events occurred at a latency of 1–2.8 years, with an 18% rate of grade 5 pulmonary toxicity [110].

Veliparib is the only agent of this class to have been tested in a randomized trial. In SWOG S1206, dose-limiting grade 3 esophagitis emerged at a low dose level; no excess toxicity over a placebo was observed, but accrual was stopped once consolidation Durvalumab became the standard of care, leaving efficacy formally uninterpretable [111]. The only phase III evaluation of a DDR inhibitor in this population, the KEYLYNK-012 trial, addresses a different question: Olaparib is added to Pembrolizumab as consolidation after concurrent chemoradiotherapy rather than delivered during irradiation and therefore tests PARP inhibition as an immunotherapy partner rather than as a radiosensitizer [112]. Its results are awaited, although the failure of the same pairing in phase III trials in metastatic NSCLC tempers expectations [113].

The CONCORDE platform is evaluating Olaparib, the PARP1-selective agent Saruparib, the ATM inhibitor AZD1390 and the ATR inhibitor Ceralasertib on a uniform 60 Gy/30 fraction backbone, with consolidation Durvalumab in two arms [114,115,116]. This trial aims to find the optimal dose and safety profile of these novel combinations. According to its first interim report, the AZD1390 arm was closed early by the independent Safety Review Committee because of excess esophageal toxicity [117], while other arms are recruiting, as they have been successfully escalated to dose levels 2 or 3 with integration of consolidation Durvalumab in two arms. Notably, although the combination of Ceralasertib and Durvalumab has yielded encouraging signals in metastatic disease [118], its role together with RT in LA-NSCLC, a mechanistically distinct setting, remains to be established [119].

Beyond these agents, the evidence is markedly thinner: the DNA-PK inhibitor Peposertib was tolerable as monotherapy with modest activity and has been combined with palliative thoracic RT but has not progressed to testing in LA-NSCLC [120], while CHK1 inhibitors remain preclinical in this setting. Table 5 summarizes, for each agent, target, trial phase, radiotherapy and systemic therapy, the details, biomarker strategy, key safety findings and current status.

### 5.3. Biomarker-Guided Patient Selection Beyond PD-L1

PD-L1 remains the only biomarker with regulatory relevance in this setting [2,3,10], but it is a coarse instrument: it was not designed to predict radiosensitivity, and it is not reliable for the purpose of identifying patients who require intensification or de-escalation. Tumor mutational burden is the most immediately available complement. In a multi-institutional cohort of 328 patients treated with concurrent CRT and Durvalumab, very high PD-L1 expression (TPS ≥ 90%) and higher TMB were independently associated with prolonged disease control [121]. In the prospective DART cohort, high blood-based TMB, PD-L1 ≥ 1% and absence of STK11, KEAP1 or NFE2L2 alterations were associated with longer PFS [122]. TMB may therefore refine, rather than replace, PD-L1, although cut-offs remain unvalidated and tissue–blood concordance is modest.

DDR status is the natural candidate biomarker for the agents discussed above, and its trajectory is instructive: the ATM-based enrichment hypothesis of the phase II HUDSON study [118] was not confirmed in the phase III LATIFY trial [123]. The reason for this discrepancy may have been due to the fact that sequencing-based calls of DDR alterations are an imperfect surrogate for functional deficiency. Functional readouts such as ATM protein loss by immunohistochemistry, RAD51 foci or SLFN11 expression may perform better and therefore their value should be tested prospectively.

DDR alterations may nevertheless carry dual value, since DDR co-mutations correlate with higher TMB and with benefit from checkpoint blockade [124]. For the same reason, the integrity of the cGAS–STING axis is a plausible predictive marker, since STING-dependent interferon production is required for the antitumor effect of RT in preclinical models [109] and is amplified by DDR inhibition [108], whereas the tumor-intrinsic silencing of cGAS or STING, frequent in lung cancer, would be expected to abrogate it. However, no analytically validated assay is currently available.

Among candidate biomarkers, ctDNA-based molecular residual disease is the closest to clinical applicability and is the only one addressing escalation and de-escalation simultaneously. Patients with detectable ctDNA after CRT derived significant benefit from consolidation immunotherapy, whereas those without residual disease did not [125]. CtDNA detected before, during or after six months of consolidation is strongly associated with inferior outcomes [126], and persistence within four months of CRT was associated with markedly shorter PFS in the DART cohort [122]. Two complementary strategies are therefore under prospective evaluation: intensification in patients with residual disease, as in ADAPT-C, where detectable ctDNA after two cycles of Durvalumab triggers the addition of chemotherapy; and shortening of consolidation, in patients with sustained molecular clearance.

## 6. Conclusions

**Figure 3:**
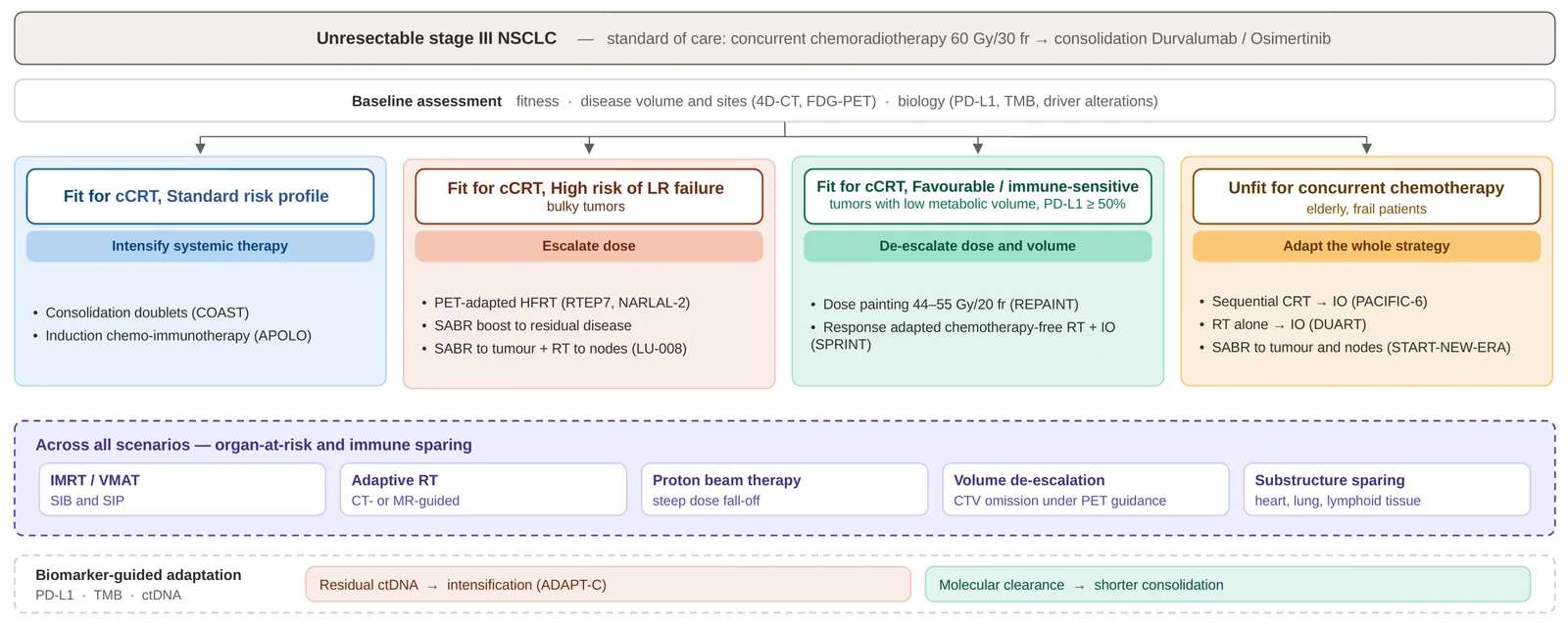
Innovative radiation therapy strategies across the clinical scenarios of unresectable locally advanced NSCLC. Following a baseline multidisciplinary assessment of fitness, disease burden and tumor biology, four scenarios can be identified. Patients fit for concurrent chemoradiotherapy with a standard risk profile are candidates for systemic intensification; those at high risk of loco-regional failure for dose escalation through hypofractionation or SABR; patients with favorable, immune-sensitive disease for risk-adapted dose and volume de-escalation; and patients unfit for concurrent chemoradiotherapy for sequential RT or RT alone with ablative or altered schedules. Advanced technology for organ-at-risk and immune sparing, including target volume de-escalation, applies across all scenarios. Finally, biomarker-guided adaptation may direct intensification or de-escalation. Abbreviations: 4D-CT, four-dimensional computed tomography; cCRT, concurrent chemoradiotherapy; CRT, chemoradiotherapy; CT, computed tomography; ctDNA, circulating tumor DNA; CTV, clinical target volume; FDG-PET, fluorodeoxyglucose positron emission tomography; fr, fraction; HFRT, hypofractionated radiotherapy; IMRT, intensity-modulated radiotherapy; IO, immunotherapy; LR, loco-regional; MR, magnetic resonance; NSCLC, non-small cell lung cancer; PD-L1, programmed death-ligand 1; RT, radiotherapy; SABR, stereotactic ablative body radiotherapy; SIB, simultaneous integrated boost; SIP, simultaneous integrated protection; TMB, tumor mutational burden; VMAT, volumetric modulated arc therapy.

Concomitant chemoradiotherapy (CRT) followed by Durvalumab consolidation, as established by the PACIFIC trial, currently represents the standard of care for unresectable locally advanced non-small cell lung cancer (LA-NSCLC) [2]. The future of LA-NSCLC treatment is driven by two main strategies (Figure 3). First, novel combinations of immunotherapy and new agents with standard CRT may provide meaningful survival benefit by enhancing the synergistic integration of local and systemic therapy. Second, to optimize patient outcomes, radiation therapy is increasingly transitioning toward a highly personalized approach that carefully balances therapeutic intensification with strategic de-escalation. Targeted dose escalation through hypofractionation (HFRT) or stereotactic ablative body radiotherapy (SABR) offers a promising strategy to improve survival by optimizing local control. Conversely, risk-adapted volume and dose de-escalation represent an alternative strategy for frail or elderly patients or those at elevated risk of severe adverse events. Furthermore, the implementation of advanced delivery techniques, such as adaptive radiotherapy and proton beam therapy, may improve critical organ-at-risk sparing by minimizing cardiac and pulmonary exposure and mitigating radiation-induced lymphopenia. The next paradigm in LA-NSCLC treatment will rely on biomarker-guided patient selection that integrates circulating tumor DNA, tumor mutational burden, and advanced imaging to accurately match each patient with the optimal combination of radiotherapy and novel systemic therapy. This precision approach is crucial for maximizing curative potential while preserving overall quality of life.

## Figures and Tables

**Table 3:** Summary of relevant stereotactic ablative body radiotherapy (SABR) studies for LA-NSCLC.

Trial, Author, Year	START-NEW-ERA, 2023, 2025 [36,37]	Kubicek et al., 2022 [43]	Parisi et al., 2019 [42]	Cong et al., 2019 [41]	Narita et al., 2019 [40]	Eriguchi et al., 2016 [39]
Design	Phase II	Phase II	Phase II	R	R	R
SABR technique	VMAT (SIB/SIP)	CK (SIB)	Tomotherapy (SIB)	CK	VMAT	VMAT
Number of patients	50	22	17	51	70	19
Median age (years)	73	67	58	63	81	79
Median follow-up (months)	72	23.1	NS	17	28.6	25
Stage IIIA/IIIB (%)	40/34	59/18	12/88	NS	17/0	NA
N2/N3 (%)	54/12	59/9	65/35	33/33	0/0	0/0
SABR/CHT-SABR/CHT–SABR–immunotherapy (%)	46%/40%/14%	0%/100%/0%	0%/100%/0%	NS	100/0/0	100/0/0
Total dose in 5 fractions (gray)	45 (T) 40 (N)	50–60 (T) 40–50 (N)	30 (T) 25 (N)	35	40/50	40/50
Median BED10 (gray)	85.5 (T) 72 (N)	100 (T) 85.5 (N)	48 (T) 37.5 (N)	59.5	72/100	72/100
LC	1 y 88%; 3 y 64%; 5 y 64%	1 y 100%	77%	1 y 54%; 3 y 40%	3 y 67.5%	2 y 85%
PFS	1 y 56%; 3 y 26%; 5 y 26%	16 months (median)	19.8 months (local recurrence); 9.7 months (distant progression)	8 months (median)	3 y 29.4% (T3); 3 y 33.3% (T4)	NA
OS	1 y 94%; 3 y 70%; 5 y 46%	1 y 82%; 2 y 53%	1 y 59%; 3 y 29%	1 y 76.5%; 3 y 20.6%	3 y 39.5% (T3); 3 y 41.7% (T4)	2 y 61%
Toxicity ≥ G3/G5	0%	9%/4%	24%/4%	10%4%	4%/1%	5%

R, retrospective; SABR, stereotactic ablative body radiotherapy; CK, CyberKnife; VMAT, volumetric modulated arc therapy; SIB, simultaneous integrated boost; SIP, simultaneous integrated protection; CHT, chemotherapy; T, primary tumor; N, involved mediastinal node; BED10, biologically effective dose; LC, local control; PFS, progression-free survival; OS, overall survival; G, grade; NA, not available.

**Table 4:** Main trials investigating CTV omission.

Study	Cohort	Prescription & Modality	4DCT	Margin Recipe	Outcomes
Liang et al. 2015 [54]	105/53 NSCLC patients with/without CTV	54–63 Gy, 27–35 fractions, IMRT	No	- CTV = GTV + 6/8 mm (ADC/SCC) - ITV = CTV + 3–15 mm - PTV = ITV + 5 mm	- No significant difference between LR, DM rate, G3-G4 esophagitis and hematological toxicity - Significant decrease in G3-G4 RP in CTV-free arm (6% vs. 1%)
Kilburn et al. 2016 [53]	110	45–74 Gy, 1.5–2.7 Gy/fraction, IMRT	Yes	- ITV defined on MIP - PTV = ITV + 5 mm - PTV expanded by 10 mm to form retrospective CTV treatment volume	- Only 2 of 22 failure events occurred in the region that would have been covered by the use of a CTV margin - 86% of local failures occurred within (CTV-free) PTV
Cui et al. 2023 [55]	45/45 NSCLC patients with/without CTV	60 Gy, 2 Gy/fraction, IMRT	Yes	- CTV = GTV + 6 mm and whole involved nodal region in case of positive lymph nodes	- No significant difference between 1 Y PFS, tumor response, LR, OS, failure pattern - Omission of CTV significantly reduced the incidence of severe respiratory events and esophagitis

NSCLC, non-small cell lung cancer; ADC, adenocarcinoma; SCC, squamous cell carcinoma; IMRT, intensity-modulated radiation therapy; CTV, clinical target volume; GTV, gross tumor volume; ITV, internal target volume; PTV, planning target volume; mm, millimeters; LR, local relapse; DM, distant metastases; MIP, maximum intensity projection; RP, radiation pneumonitis; PFS, progression-free survival; OS, overall survival; G, grade; 4DCT, four-dimensional computed tomography.

**Table 5:** DNA damage response (DDR) inhibitors combined with radiotherapy in NSCLC: target, trial phase, radiotherapy and systemic therapy details, biomarker strategy, safety findings and current status.

Agent	Target/Mechanism	Trial (Phase)	RT	Systemic Therapy	Biomarker Strategy	Key Safety Findings	Current Status
Olaparib	PARP1/2 trapping; blocks SSB repair, generating replication-associated DSBs	CONCORDE-A (phase Ib, NCT04550104); NKI phase I (NCT01562210)	CONCORDE: 60 Gy/30 fr. NKI: 66 Gy/24 fr	CONCORDE: concurrent Olaparib. NKI: ± concurrent daily low-dose cisplatin (6 mg/m^2^)	None	NKI: MTD only 25 mg once daily without cisplatin; severe pulmonary AEs at 1–2.8 y latency, 18% grade 5	CONCORDE-A escalated to dose level 3; no efficacy data
Veliparib	PARP1/2 (weak trapping)	SWOG S1206/8811 (phase I → randomized, placebo-controlled phase II)	Conventionally fractionated thoracic CRT	Concurrent weekly carboplatin–paclitaxel + 2 consolidation cycles	None	DLTs: grade 3 esophagitis at 40 and 80 mg; no excess toxicity vs. placebo	Closed prematurely once Durvalumab became SOC; efficacy uninterpretable
Saruparib (AZD5305)	PARP1-selective trapping (spares PARP2)	CONCORDE-E (phase Ib)	60 Gy/30 fr	Concurrent Saruparib; consolidation Durvalumab	None	Escalated to dose level 2; no DLT reported to date	Recruiting; no clinical confirmation yet
AZD1390	ATM kinase inhibition; impairs DSB signaling and repair	CONCORDE-B (phase Ib)	60 Gy/30 fr	Concurrent AZD1390	None	DLTs in 4/14 at dose level 3 (40 mg on RT days); grade 3 + esophagitis in 64% of prolonged duration (median 67 vs. 27 days with RT alone) despite comparable esophageal dose metrics	Arm closed early by independent Safety Review Committee for excess esophageal toxicity. Median PFS 9.5 vs. 8.8 months with RT alone
Ceralasertib (AZD6738)	ATR kinase inhibition; abrogates replication checkpoint; STING-mediated immune priming	CONCORDE-C (phase Ib)	CONCORDE: 60 Gy/30 fr	CONCORDE-C: concomitant Ceralasertib; consolidation Durvalumab + Ceralasertib	None	Escalated to dose level 2 with Durvalumab consolidation	Recruiting
Peposertib (M3814)	DNA-PK inhibition; blocks non-homologous end joining	NCT02516813 (phase Ia/Ib)	Palliative 30 Gy/10 fr (thorax)	Peposertinib	None	DLTs during escalation with RT	No mature data in LA-NSCLC; a stage IIIA/B expansion cohort was planned but development has not progressed to randomized testing

AE, adverse event; ATM, ataxia-telangiectasia mutated; ATR, ataxia-telangiectasia and Rad3-related; CRT, chemoradiotherapy; DDR, DNA damage response; DLT, dose-limiting toxicity; DNA-PK, DNA-dependent protein kinase; DSB, double-strand break; fr, fraction; LA-NSCLC, locally advanced non-small cell lung cancer; MTD, maximum tolerated dose; NKI, Netherlands Cancer Institute; PARP, poly (ADP-ribose) polymerase; RT, radiotherapy; SOC, standard of care; SSB, single-strand break; STING, stimulator of interferon genes.

## Data Availability

No new data were created or analyzed in this study. Data sharing is not applicable to this article.
